# 3D printed zirconia used as dental materials: a critical review

**DOI:** 10.1186/s13036-023-00396-y

**Published:** 2023-12-21

**Authors:** Guanyu Su, Yushi Zhang, Chunyu Jin, Qiyue Zhang, Jiarui Lu, Zengqian Liu, Qiang Wang, Xue Zhang, Jia Ma

**Affiliations:** 1 Liaoning Provincial Key Laboratory of Oral Diseases, School and Hospital of Stomatology, China Medical University, No. 117 Nanjing North Street, Shenyang, 110001 China; 2 Department of Orthodontics, School and Hospital of Stomatology, China Medical University, No. 117 Nanjing North Street, Shenyang, 110001 China; 3 Shi-Changxu Innovation Center for Advanced Materials, Institute of Metal Research, Chinese Academy of Sciences, Shenyang, 110016 China; 4 School of Materials Science and Engineering, University of Science and Technology of China, Hefei, 230026 China

**Keywords:** 3D printing, Zirconia, Dental prosthesis, Implant, Maxillofacial surgery

## Abstract

In view of its high mechanical performance, outstanding aesthetic qualities, and biological stability, zirconia has been widely used in the fields of dentistry. Due to its potential to produce suitable advanced configurations and structures for a number of medical applications, especially personalized created devices, ceramic additive manufacturing (AM) has been attracting a great deal of attention in recent years. AM zirconia hews out infinite possibilities that are otherwise barely possible with traditional processes thanks to its freedom and efficiency. In the review, AM zirconia’s physical and adhesive characteristics, accuracy, biocompatibility, as well as their clinical applications have been reviewed. Here, we highlight the accuracy and biocompatibility of 3D printed zirconia. Also, current obstacles and a forecast of AM zirconia for its development and improvement have been covered. In summary, this review offers a description of the basic characteristics of AM zirconia materials intended for oral medicine. Furthermore, it provides a generally novel and fundamental basis for the utilization of 3D printed zirconia in dentistry.

## Introduction

Yttria-stabilized tetragonal zirconia polycrystal (Y-TZP) is currently almost the most durable restorative ceramic available. Their exceptional mechanical qualities, along with their biocompatibility and corrosion resistance, are what draw individuals especially [1–4]. However, the means of manufacturing ceramics with required properties and complicated geometries is still with high cost and ineffectiveness [5]. Traditional zirconia processing methods are usually subtractive manufacturing (SM). SM zirconia materials still have drawbacks in their applications. CAD-CAM subtractive manufacturing results in a large amount of waste of raw materials leading to a reduction of production efficiency [6]. Moreover, the milling and postprocessing such as surface treatment steps may create microcracks to the material which has an adverse effect on mechanical properties of final products [7, 8]. Furthermore, it is noteworthy that SM is difficult to satisfy patients’ personalized needs with the popularization of bionics consciousness. These constraints have driven researchers to hunt for better-performing approaches of fabricating zirconia materials.

**Fig. 1:**
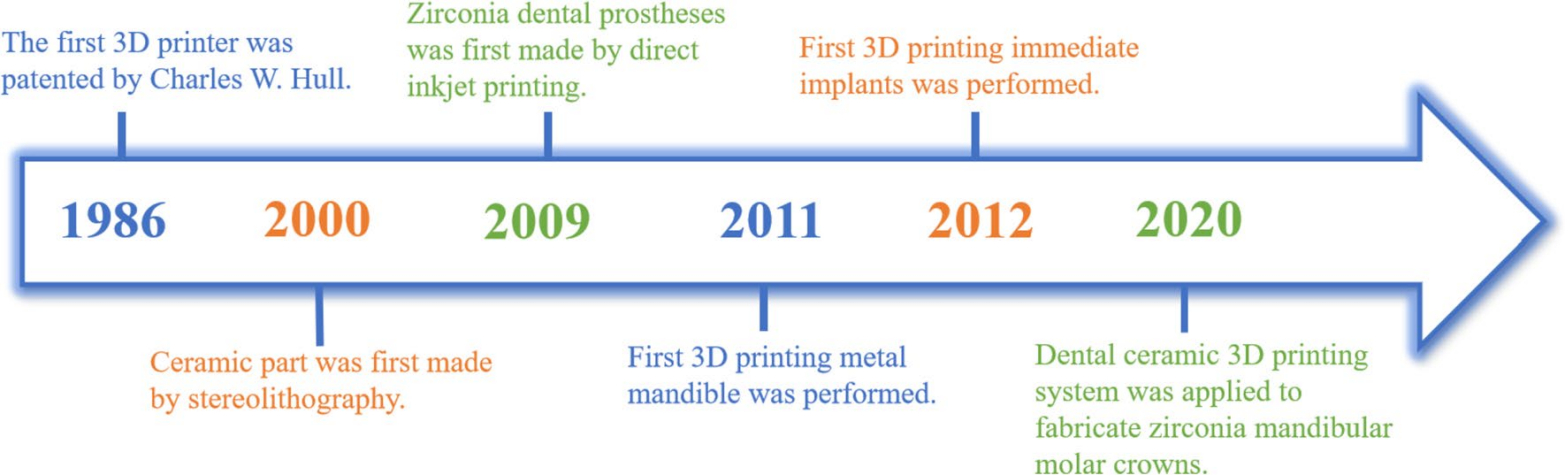
The historical process related to 3D printing in dentistry

The first three-dimensional (3D) printer was patented by Charles W. Hull in 1986. From then on, 3D printing has expanded rapidly as an industrial technology over its near 40-year history [9, 10]. In 2000, ceramic parts were firstly made by stereolithography [11]. For dentistry, zirconia dental prostheses were first made by direct inkjet printing in 2009 [12]. The first metal mandible was produced in 2011 [13]. In 2012, root analogue implant was manufactured for immediate implant [14]. The LCM (Lithography-based Ceramic Manufacturing) process used by Lithoz (Vienna, Austria) offers a cutting-edge method dedicated for dental zirconia. Crowns for the mandibular molars were manufactured using Lithoz’s LCM technology [15] (Fig. 1). The additive manufacturing (AM) method has been applied in a wide range of domains, including dentistry and personalized medicine [16]. Fused deposition modeling, stereolithography (SLA), selective laser sintering, photopolymer jetting, inkjet printing, and powder binder printing are representatives of additive manufacturing technologies [17–20]. For additive manufacturing, a wide range of materials are employed, including composites, polymers, metal alloys, and ceramic [19–22]. Aiming towards more customized (multi-material, multi-shade/translucency) restorations, 3D printing is currently on the rise [23]. Nevertheless, 3D printed zirconia dental materials can be roughly regarded as still being in its infancy, and numerous researchers are working on the performance of 3D printing zirconia materials and comparing them with CAD/CAM ones. It is significant to consider whether they can be widely employed in oral medicine and clinical indications of different materials. Unfortunately, despite the fact that an increasing number of research has focused on the characteristics of AM dental zirconia materials, a thorough assessment of AM zirconia is still lacking.

This review gives an overall summary of the current research progress on AM dental zirconia materials. Four aspects are elaborated here: the physical and adhesive properties, accuracy and biocompatibility of AM zirconia materials, their clinical applications, challenges and optimization methods of AM zirconia and its perspective. It aims to give guidance and fundamental support for upcoming research to create new material types. More importantly, it provides a basis for how clinicians choose different materials.

## Properties of AM zirconia

### Microstructures

As is known, the microstructure of a material determines its physical and chemical properties. On one hand, the toughness of a material depends on the microscopic phase composition. AM zirconia, like milled zirconia, consists of tetragonal phase only. Its constituent particles are evenly distributed and the grain size is about 0.6 μm. There is no significant difference of grain size, crystalline phase structure between digital light processing (DLP) zirconia and milled one [8].

For Y-TZP, the high-temperature phase (tetragonal phase) of zirconia may be maintained at normal temperature by adding yttrium oxide with Y-TZP. High strength is found in the tetragonal phase (t) and zirconia’s metastable tetragonal phase quickly changes into monoclinic phase (m) under specific stress. This transformation is followed by a 3–5% volume expansion namely transformation toughening. However, in humid settings, zirconia’s tetragonal (t) phase can change spontaneously into monoclinic (m). Intergranular microcracking is a side effect of what is known as aging, hydrothermal degradation, or low-temperature degradation (LTD), which causes the loss of mechanical characteristics [24]. LTD has no effect on surface roughness, but it affects the polarity and surface energy of the material, which will make a difference on biocompatibility [25, 26]. One recent study has shown that after aging (after both 5 and 10 h) the m-phase of DLP one was higher than that of SM one. That might because the composition of the two were different before aging. Besides, the t / m transformation rate of zirconia produced by SLA is significantly faster than that of traditional SM one [24]. What’s more, cubic grains contain a lot of yttrium, which might cause yttrium depletion in nearby tetragonal grains as they age. Additionally, cubic grains act as the nucleation sites for the phase transition from tetragonal to monoclinic phase [27]. Research reported that adding alumina could weaken the LTD effect of Y-TZP [28].

On the other hand, the surface roughness of dental zirconia ceramics is crucial for predicting its service reliability. High surface roughness of dental prosthesis means that plaque accumulates on the surface of prosthesis, which might cause secondary caries and periodontitis [29]. Roughness less than 0.58 µm is considered clinically acceptable [30]. However, a previous study showed the surface roughness did not meet the standard with the minimum value of 0.71 μm [31]. This may be explained by the stepping effect of 3D printing process.

Micropores and agglomerates might affect the mechanical properties of materials. The surface microstructure of AM zirconia was observed and it was found that there were micropores ranged from 196 nm to 3.3 µm [32]. Other research also noted the presence of pores on the surface of AM zirconia [33]. The presence of agglomerates affects the roughness and the aesthetics of the material. It is believed that the appearance of the pores is due to the weak bonding between the layers of the printed material and the uneven slurry. In principle, agglomerates are more likely to form when the slurry is thicker [34].

### Mechanical properties

Numerous experiments have been conducted to evaluate the mechanical properties of 3D printed zirconia (Table 1). Vickers hardness characterizes the strength and toughness of materials, which means the durability of the material. The experimental results indicated that the Vickers hardness of 3D printed zirconia materials can be up to 13.4 ± 0.2 GPa. Researchers tested apparent hardness and true hardness using proportional specimen resistance (PSR) and modified proportional specimen resistance (MPSR) models. It came out that the hardness results were evidently load-dependent in terms of hardness. Both apparent and true hardness of 3D printed materials were lower than milled ones [35]. Vickers hardness was noted to be strongly dependent on pore size and porosity, and these factors were observed in the experiment [35].

**Table 1:** Mechanical properties of 3D printed zirconia applied in dental materials

**Raw Materials**	**Printing Methods**	**Printing Systems**	**Printing Parameters**	**Mechanical Properties**	**Reference**
Enamel	-	-	-	Vickers Hardness: 2940–4800 MPa Fracture toughness: 0.6–1.8 MPa·m^1/2^ Compressive strength: 261–400 MPa	
Dentin	-	-	-	Vickers Hardness: 570–600 MPa Fracture toughness: 3.1 MPa.m^1/2^ Compressive strength: 232–305 MPa	
58 vol% Y-TZP	DLP	Ceramatrix, QuickDemos Company, China	Layer thickness: 25 µm Light intensity: 90 mW/cm^2^	Hardness: 1193 HV Fracture toughness: 3.44 ± 0.23 MPa·m^1/2^	[35]
58 vol% Y-TZP	DLP	QuickDemos Company	Layer thickness: 25 µm Light intensity: 90 mW/cm^2^	Uniaxial strength: 1004.4 MPa Biaxial strength: 741.8 MPa	[8]
TZ-3YS-E	LCM	ADMAFLEX 2.0; ADMATEC Europe BV, The Netherlands	Layer thickness: 30 µm	Flexural strength: 943.26 ± 152.75 MPa Weibull modulus: 7.032	[36]
60 vol% Y-TZP	MEX	Delta Wasp 2040 Turbo, Wasproject, Massa Lombarda, IT	Layer thickness: 0.6 mm, Vertical wall thickness: 2.4 mm, Printing speed: 60 mm/s, Infill density: 65%, Pressure: 0.6 MPa	Flexural strength: 488.96 ± 79.84 MPa Fracture toughness: 2.63 ± 0.2 MPa·m^1/2^ Compressive strength: 1.56 GPa Vickers hardness: 11.52 ± 0.57 GPa	[37]
3DCeram Sinto	SLA	3DCeram Sinto	Layer thickness: 25 µm	Weibull biaxial strength: 1108.8 MPa	[38]
42 vol% Y-TZP	LCM	CeraFab 7500 printer	Layer thickness: 25 μm; Exposure time: between 2.2 and 2.7 s	Vickers hardness: 13.4 ± 0.2 GPa Fracture toughness: 5.1 ± 0.3 MPa·m^1/2^ Flexural strength: 878 MPa	[34]

Another most evaluated mechanical property is flexural strength. The average bending strength of AM ceramics meets the level 5 standard in ISO 6872 (> 800 MPa) [39]. Although the Weibull modulus of the zirconia produced by DLP is lower than that of CAD/CAM zirconia as indicated by uniaxial (three-point bending) and biaxial (ring on ring) tests, studies have found that the zirconia specimen acquired favorable flexural strength close to that of a conventional CAD/CAM one [8]. While higher flexural strength was acquired by 3D printing than CAD/CAM [36]. Milled zirconia blocks are more resistant to uniaxial compression than 3D printed blocks. However, the 3D printed samples without fracture show interesting characteristics, such as better elastic modulus and lower compression deformation tendency compared with the milled samples [40].

Awareness of the various ways that a prosthesis could malfunction is necessary for the investigation of dental ceramic optimization. Dental zirconia fails, like all ceramic materials, through brittle fracture that results from flaws that appear after treatment or during use. They are dispersed throughout the material and come in a variety of sizes and shapes, usually being very small [41]. A majority of studies found no significant variation in fracture toughness between 3D printed and CAD/CAM zirconia parts [28, 42]. While certain experimental findings indicated that 3D printed zirconia had poorer fracture toughness than CAD/CAM [34]. Studies showed that compared with zirconia alone, zirconia toughened with alumina has higher fracture toughness [28].

There are studies on the effect of aging on the mechanical properties of 3D printed zirconia materials. It turned out that the m-phase content for SLA and DLP increased with the aging time, whereas the mechanical properties did not significantly decrease, indicating the stability of both materials [24]. Another research was conducted in a chewing simulator with extra cyclic temperature fluctuations between 5 and 55 °C for 5 million cycles in water, and it turned out that there was no discernible difference in the bending moment after fatigue with regard to the long-term durability of AM implants. The implants exhibited a considerably larger fracture stress and bending moment during mechanical fatigue in water at 90 °C. When mechanical wear and tear in a chewing simulator with water at 90 °C was combined, 31 vol% t-ZrO_2_ at the surface experienced tetragonal-to-monoclinic phase change, including wear and tear and aging [43].

In general, solid amount, printing parameters, debinding and sintering procedures impact the performance of the final products prominently. Higher solid amount means better mechanical performance. Researchers observed significant differences in the indentation fracture resistance with different building orientations. In particular, the performance has been improved when the layer line orientations were 45° inclined to the indentation direction [42]. Poor temperature control during the sintering process leads to high porosity of the product [44].

### Adhesion properties

Zirconia is an inert material. Durable resin-ceramic adhesion, which has been one of the difficult difficulties in dentistry, may affect the clinical outcome of ceramic restorations. Researchers observed the formation of dimples between the combination interfaces. They analyzed that this is due to the gradual cracking of the ceramic interface due to the absorption of external stress and hypothesized that dimples represent a strong bonding force between the two. This results in better bonding of 3D printing zirconia than the CAD/CAM one [45]. A Schwickerath adhesion test on DLP zirconia cope and it turned out that printed zirconia specimen exhibited a similar adhesive performance as CAD/CAM ones [46].

### Accuracy

Accuracy is a key indicator for evaluating the performance of the final product by AM. For dental applications, accuracy enhancement of AM technology is still necessary. According to the International Organization for Standardization (ISO), ‘accuracy’ is the degree to which the measured value of an object and its real value agree (ISO/IEC GUIDE 99:2007 (E/F)). The term ‘accuracy’ is defined to include two parameters: “trueness”, which informs us of the divergence from the true value, and ‘precision’, which informs us of the consistency of repeated measurements [47]. It is well knowledge that improved fabrication precision results in better prosthetic fit. High accuracy means less clinical adjustment time. More importantly, less adjustment can reduce the damage caused by the operation and improve the mechanical quality of the products. As for implants, poor accuracy would lead to plaque accumulation, gingival inflammation and microleakage [48].

**Fig. 2:**
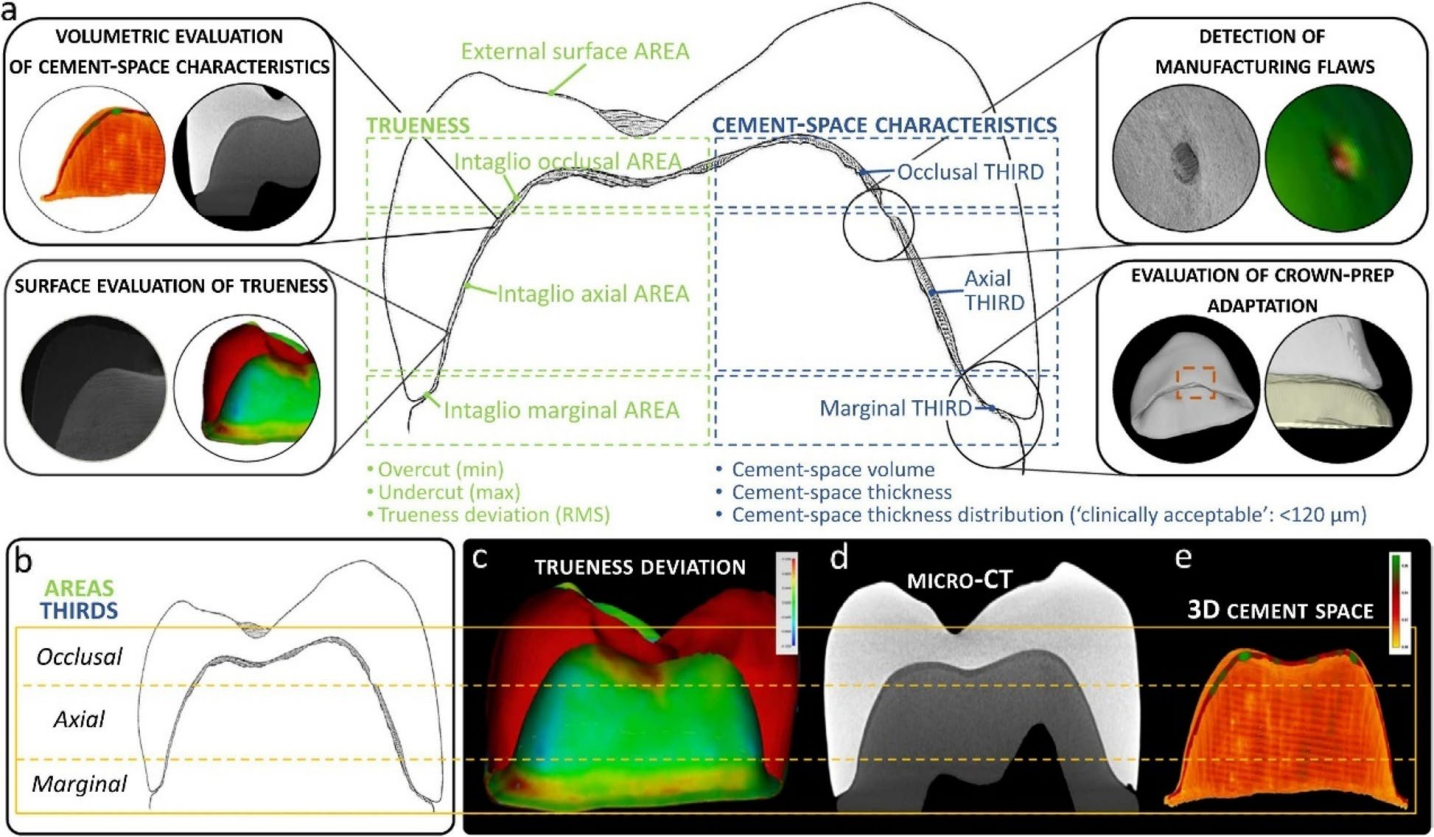
Schematic illustrating the different evaluation methodologies (four rounded squares) and the two study parameters TRUENESS, as determined in terms of the overcut (min), undercut (max) and trueness deviation (RMS) indices at the intaglio occlusal, axial and marginal areas, and the external surface area, and CEMENT-SPACE CHARACTERISTICS, as determined in terms of cement-space volume, thickness and thickness distribution at the occlusal, axial and marginal thirds in (**a**) and (**b**), with a color map representing trueness deviation in (**c**), a micro-CT cross-section in (**d**) and a 3D color map of cement-space thickness distribution in (**e**) [49]

To evaluate the trueness of 3D printed crowns, researchers divided dental crowns into inner and outer surfaces, focusing on exploring the characteristics of the inner surface and the thickness and corresponding volume of the cement space. The crown was divided into three parts: marginal, axial and occlusal area in the axial orientation. They used visible-light scan and Micro-CT to obtain three-dimensional data and compare them with the original CAD design. As a result, volumetric evaluation of cement space, surface evaluation, manufacturing flaws and crown- prep adaptation were obtained (Fig. 2). The occlusal region of the intaglio can be expected to have the least optimal trueness and crown adaption for monolithic zirconia crowns for both SM and AM manufacturing techniques, according to a study [49]. Therefore, intaglio’s marginal area might be the determining factor recorded between 3D printing and milling. Researchers compared the two parties at the same time: milled ones and 3D printed ones (Table 2). While the intaglio occlusal surface, intaglio surface, and marginal area demonstrated the opposite outcome, the printing group’s accuracy in regard to the external surface of the crown outperformed that of the CAD/CAM group. None of these findings, however, revealed statistically distinct differences [45, 50, 51]. Nevertheless, opposite results have been reported: the accuracy of AM full-contour crown was lower than that of SM only in the marginal regions. Splint zirconia crown refers to two-piece crown, which has been evaluated for better mimicking the structure of enamel and dentin. It was shown that the trueness of CAD/CAM group and AM splint zirconia group were clinically accepted besides AM zirconia group [52, 53].

**Table 2:** Trueness of 3D printed zirconia materials applied in dental materials

**Raw Materials**	**Printing Methods**	**Printing Systems**	**Printing Parameters**	**Objectives**	**Trueness (µm)**	**Reference**
45 wt% ZrO_2_ powder	DIP	Commercial Carmel 1400 (Xjet) inkjet printer	Layer height: 10.5 µm; Resolution: 16.000 × 17.625 µm	Crown	Intaglio marginal area: 97 ± 20 Intaglio axial area: 33 ± 7 Intaglio occlusal area: 127 ± 54 External surface area: 64 ± 12	[49]
INNI-Cera, AON, Gunpo, Korea	DLP	AON, Gunpo, Korea	-	Crown	Internal area: 40.41 ± 1.25 Marginal area: 48.75 ± 4.39	[45]
3DMix ZrO_2_ (3DCeram)	SLA	CERAMAKER 900; 3DCeram Co	-	Crown	Internal area: 85.0 ± 48 Marginal area: 79.5 ± 49.2	[52]
3DMIXZrO_2_L; 3DCeram Co	SLA	CERAMAKER 900; 3DCeram Co	-	Crown	External area: 53 ± 9 Intaglio area: 38 ± 12 Marginal area: 34 ± 5 Intaglio occlusal area: 27 ± 17	[50]
-	LCM	CerafabS65®, Lithoz	-	Crown	All crown: 33.2 ± 1.0 Marginal area: 22.8 ± 1.6 Occlusal area: 38.9 ± 2.4	[53]
45 wt% ZrO_2_ powder	SLA	CSL 150, Porimy	Layer thickness: 25 µm	Crown	Occlusal area: 63.4 ± 6.54 Axial wall area: 135.08 ± 10.55 Marginal area: 169.58 ± 18.13	[54]
-	DLP	ADMATEC Europe BV, Moergestel, the Netherlands	-	Implant	RMS (root mean square): 150 ± 99	[55]
-	DLP	ADMAFLEX 2.0; ADMATEC Europe BV, The Netherlands	Resolution: 1080 × 1920 pixels Pixel size: 49.61 µm Layer thickness: 30 µm	Implant	RMS: 100 The average deviation: 89 and 129 ± 68 homogenously distributed along the length.	[32]

Understanding the precision of fabrication, its systematic and haphazard faults, as well as the constraints that could affect the proposed and accomplished restoration, is crucial. What’s more, printing technique and parameters make a difference. Studies found that reducing the layer height can increase the accuracy of the final product [56]. This may be explained by the surface stepping phenomenon of AM process [57]. Similar results were also detected in another four-unit bridge experiment. Researchers stressed that the accuracy of DLP was lower than SLA [58]. The accuracy is different under different porosity, which should be considered before manufacturing and adjusted by sintering [59]. Research showed that through some special process like using carefully designed porous polymer molds, 3D printed flexible products can be accurately shaped to a certain curvature [60]. Some models and methods can be adopted to predict the shrinkage of samples for better control [61]. Besides, both the tooth types and the angle of construction affect the accuracy of the products.

### Biocompatibility

Implants and scaffolds require intricately interconnected cellular structures, which 3D printing technology can create. These structures can encourage the production of bone from the surrounding tissue [62]. Zr-based materials have shown promising outcomes with low ion release, low cytotoxicity, favorable biocompatibility, and good osseointegration capability [48]. 3D printed zirconia composite as implants and scaffolds has become a promising research direction (Table 3).

**Table 3:** Biocompatibility of 3D printing zirconia scaffolds

**Raw materials**	**Surface Treatment**	**In vivo or in vitro**	**Cells or animals tested**	**Outcomes**	**Reference**
ZrO_2_/SiO_2_	-	In vitro	MG63	50wt% zirconia powder group showed the best proliferation.	[62]
PCL/ZrO_2_	-	In vitro	MC3T3	The PCL/ZrO_2_ composite scaffold group showed better cell adhesion, proliferation and growth and showed better ALP activity and accommodated more effective bone mineralization.	[63]
BCP/ZrO_2_	-	In vitro	MG63 hMSCs	BCP/ZrO_2_ scaffold had a good biocompatible property on proliferation of MG63 cells and promoted osteogenic differentiation.	[64]
HAP/ZrO_2_	-	In vitro	mBMSCs	Stem cells adhered, grew, and proliferated on HAP/ZrO_2_. HAP/ZrO_2_ ceramics had good porosity, high surface roughness, and are easy for cells to climb.	[65]
ZrO_2_/CS	-	In vitro	MC3T3-E1	Scaffolds doped with more CS possessed better biological activity and were more beneficial to MC3T3-E1 cells proliferation and differentiation.	[66]
ZrO_2_/RGO/HA	-	In vitro	AD-MSCs	HA and GO had a more beneficial presence to reduce cytotoxicity than individual presence.	[67]
ZrO_2_	Zn-HA/glass	In vitro	DPCs	The composite constructs exhibited superior cell-adhesion, distribution, and osteogenic differentiation ability.	[68]
ZrO_2_	HA/CS	In vitro	MC3T3-E1	CS/HA coating on the surface of zirconia scaffold had a positive effect on the proliferation of MC3T3-E1 cells.	[69]
ZrO_2_	ZnO	In vivo	New Zealand white rabbits	HE staining results indicate mild inflammatory response. ZrO_2_–ZnO ceramics had good biocompatibility when contacting bone tissue and surrounding muscle tissue.	[70]
ZrO_2_	-	In vivo	Rat (PDGFRs, osterix, osteopontinand osteocalcin	The bone formation has been considerably enriched in GBR sites using 3D printed zirconia barrier.	[71]

**Fig. 3:**
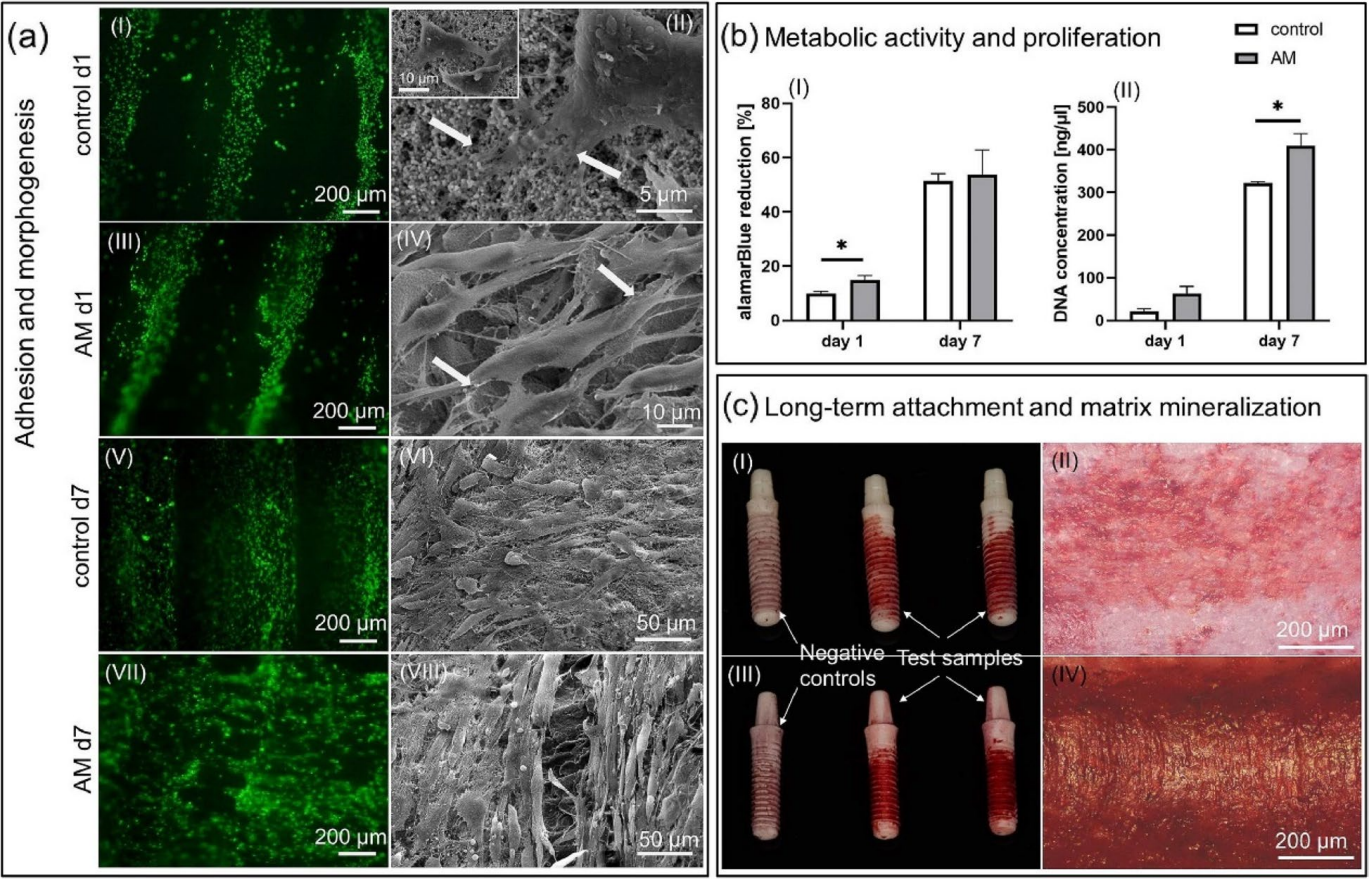
Cell biological evaluation with primary human osteoblasts. **a** Analysis of cell adhesion and morphogenesis by fluorescence microscopy and SEM. **b** Data obtained in the metabolic alamarBlue assay and by DNA quantification; statistically significant differences are marked with bars above the corresponding columns. **c** Photographs and micrographs of the AlizarinRed staining. Cells on the negative controls were cultivated in standard culture medium, while the cells on the test samples in osteogenic medium [43]

Various in vitro investigations have established the biocompatibility and osteogenic potential of 3D printed zirconia composite. Long-term attachment, osteoblast proliferation, and matrix mineralization are made achievable by fabricating direct implant shape with distinctive scattering geometries and surface topographies. These result in an elongated osteoblast morphology with uniform cell orientation. Fluorescence and scanning electron microscopy are used to analyze the cell distribution on the implant’s surface. It turns out that the groove structure on the surface of AM implants helps cells form slender shapes and uniform cell orientation. The osteoblasts are ‘contact-guided’ by the multilayer nature of AM implants. According to the concept of ‘contact guidance’, sticky signals from the extracellular matrix (ECM) or a biomaterial govern cell adhesion, which in turn determines cell shape and arrangement [72]. The tailored surface structure of AM implants appears to be advantageous for cell metabolism and proliferation. Compared to the control implant, the density of the AM implant covered by mineralized matrix is higher, demonstrating the favorability of matrix mineralization, or the matrix is more securely fixed on the AM implant than on the control implant (Fig. 3) [43]. Studies have shown that ZrO_2_-SiO_2_ (ZrO_2_:SiO_2_ = 1:1) had the best MG63 cell affinity among the ZrO_2_-SiO_2_ composite with other ratios [48]. The biocompatibility of the ZrO_2_-CS (calcium silicate) composite scaffold is excellent. Additionally, high CS concentration is better for MC3T3-E1 cell adhesion, proliferation, and differentiation, all of which are essential for the bone repair process [68].

Through the use of melt mixing, nano zirconia powder has been incorporated into polycaprolactone (PCL) material, and a regular grid scaffold is created via 3D printing. The embedding of nanometer-sized zirconia powder is demonstrated to improve the hydrophilicity and water absorption of the composite scaffold, which is favorable for the exchange of nutrients and promotes MC3T3 cell adhesion, proliferation, and growth [63]. According to studies on the differentiation of human mesenchymal stem cells (hMSCs) on BCP/ZrO_2_ scaffolds in static and dynamic culture conditions, the expression of bone morphogenic protein-2 (BMP-2) was higher on BCP/ZrO_2_ scaffolds than on BCP scaffolds [64]. An experiment using in vitro cell culture was performed to gauge the biocompatibility of the HAP/ZrO_2_. The results showed that the aggregation behavior of mBMSCs cells on the materials was more visible owing to the roughness of the surface of composite ceramics, and the growth morphology of mBMSCs cells became more elongated [68].

At the same time, in vitro experiments using dental pulp cells (DPCs) demonstrated that Zn-HA/glass composite-coated glass infiltrated zirconia scaffolds performed satisfactory biocompatibility, including the attachment, proliferation, and osteogenic differentiation of DPCs [68]. Using 3D printing technology, porous zirconia scaffolds are created and then coated in calcium silicate and hydroxyapatite. Test results revealed that coated scaffolds had good cytocompatibility and helped MC3T3-E1 cells proliferate and differentiate [69]. Low temperature 3D printing technology can effectively avoid interference from high-temperature environments on protein activity. Applying natural phospholipid modified protein technologies with low temperature printing technology to fabricate scaffold with Bone Morphogenetic Protein-2 and Human Beta Defensin-3 (BMP2 and HBD3) has been conducted. It turns out that this scaffold promotes osteogenic differentiation [73]. Producing biocompatible surface coatings for zirconia materials in this way could become a future research direction. There are in vivo experiments evaluating the biocompatibility of 3D printed zirconia. According to recent research, HE staining results indicate mild inflammatory response of ZrO_2_–ZnO ceramics with good biocompatibility [70]. What’s more, one 3D printed GBR barrier was fabricated showing good osteogenic response and biocompatibility by evaluating the expression of PDGFRs, osterix, osteopontin and osteocalcin [71]. However, in vivo experiments on 3D printed zirconia are still lacking.

## Clinical applications

**Fig. 4:**
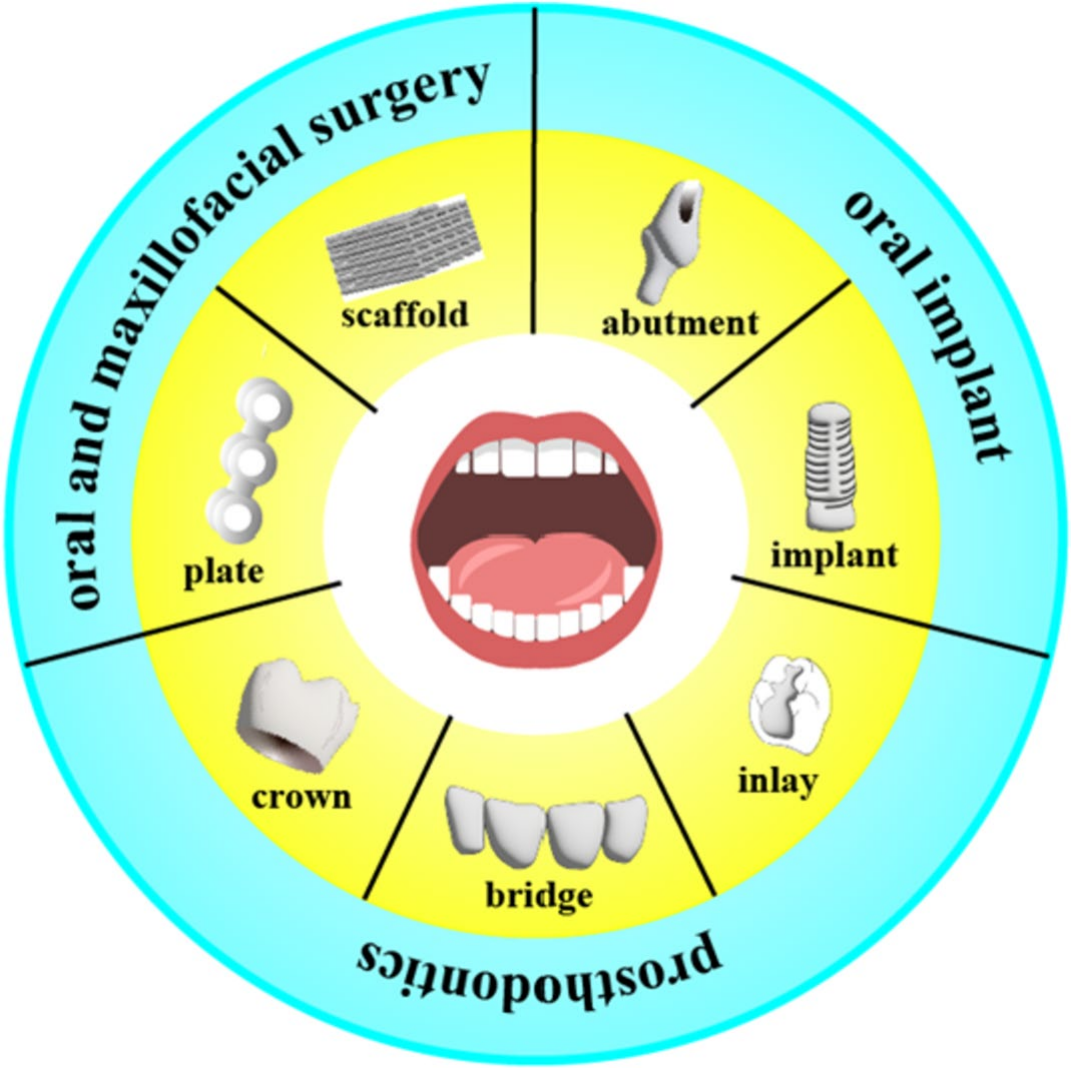
Application of 3D printed zirconia materials in the field of dentistry

3D printed zirconia ceramic is a promising candidate material for a variety of applications because it provides great qualities similar to SM zirconia. In the past 10 years, 3D printing technology has become commonly employed in dentistry, including patient care and dental education. And the experiment fields of 3D printed zirconia include prosthodontics, oral implant and oral maxillofacial surgery. Examples include the ultra-thin occlusal veneer, cope, crown and bridge, implant, abutment, scaffold and so on. The most recent developments in the application of zirconia materials for 3D printing in dentistry will be specified here (Fig. 4).

### 3D printed zirconia occlusal veneers

Ultra-thin occlusal veneer is a novel minimally invasive method that can minimize the removal of healthy tooth tissue. It is suitable for teeth that have been worn or eroded. Study showed that the load-bearing capacity of 3D printed zirconia was significantly higher than that of the CAD/CAM one when simulating the chewing environment [74]. A high degree of accuracy is also critical for printing zirconia veneers, which means that the veneers have an excellent fit to the dentin. The marginal adaptation (95 µm) and production accuracy (26 µm) of AM zirconia occlusal veneers generated via LCM (lithography-based ceramic manufacturing) are comparable to those derived from conventional methods [75]. Encouragingly, accuracy of occlusal veneer that is higher than milled ones has also been reported [76].

### 3D printed zirconia cope

The effectiveness of a zirconia cope using a pattern made by a 3D printer has been reported. Adhesion is quite essential for the application of PFZ (porcelain fused zirconia) especially for the anterior teeth, which is the key to the success of the restoration. Stable adhesion can improve the crushing resistance of the restoration. A Schwickerath adhesion test has been done on DLP zirconia cope and found that printed zirconia specimen exhibited a similar adhesive force as CAD/CAM ones [46]. Another study found that AM zirconia cope obtained higher bond strength than SM one because there were more dimples on the surface of AM one [45].

### 3D printed zirconia full-contour crown and bridges

Studies on mechanical properties and accuracy of zirconia full-contour crowns and bridges produced by 3D printers have been carried out by a number of researchers [8, 35–38]. As mentioned above, the mechanical properties and accuracy of 3D printed zirconia can meet clinical standards. Novel material composite, like PLA/ZrO_2_ synthetic resin as the material, glycerin and silane coupling agent as the binder to build dental crown samples has been conducted and obtains satisfactory results [77].

3D printed zirconia bridges have been fabricated and acquisitioned superior properties. The sintered part has an average hardness of 1224 HV, a density of 5.45 g/cm^3^, a flexural strength of 641.04 MPa and a clinically acceptable marginal gap. However, there are still shortcomings. Results showed that samples did not match the expected color, appearing purple. Besides, cracks were observed on the outer surface of the SLA samples [56].

### 3D printed zirconia implant and abutment

3D printed implants are one of the most promising applications of this technology in the sphere of dentistry, especially zirconia implant. The advantages of 3D printed zirconia implants can be analyzed from the following perspectives: first and foremost, the error can be narrowed by 3D printing ‘custom-made’ implants. A good fit of the implant to the bone tissue can greatly reduce the time required for recovery. By 3D printing, implant materials with specific porosity can be produced to finish the osseointegration process better. 3D printed zirconia porous materials are beneficial to the proliferation and adhesion of cells but have an inhibitory effect on differentiation. Second, compared to titanium implants, zirconia implants have the advantages of better aesthetic performance, which is especially noticeable in the anterior dental implants. Compared with titanium, zirconia has less bacterial adhesion and biofilm formation. Zirconia surfaces are less prone to infection [78]. Additionally, zirconia implants have several advantages over titanium implants, including no metal aura in situations when the buccal bone wall is inadequate and/or corrosion resistance, thin mucosal biotype and hypoallergenicity [79]. Traditional implants require surface treatment, such as phase transformation, chemical coating and laser treatment, which seem to be quite cumbersome. However, the significant advantage of 3D printed zirconia implants is to directly fabricate suitable porous structure to promote osseointegration for prolonged lifetime [43, 80].

A number of studies have been conducted to evaluate the clinical applicability of 3D printed zirconia implants. Research showed that the flexural strength and Weibull modulus of 3D printed implants were within clinically acceptable limits [32]. Compared to SM zirconia, AM zirconia has a smoother surface, resulting in a more random arrangement of cells on its surface [81]. AM zirconia is more affected by accelerated aging, namely greater phase transition rate (monoclinic phase increase). Furthermore, accelerated aging results in submicron changes in the surface morphology of both AM and SM zirconia [81]. These may be related to the low temperature aging (LTD) phenomenon of zirconia.

Smart implants are now visible to the general public. Sensing, actuation, and control capabilities are combined in smart implants to describe and analyze conditions and make judgments in a predictive or adaptive way to carry out intelligent activities. These implants need communication cavities, such as wires and antennas, sensors, and actuators. With the introduction of 3D printing, it has been possible to create implants that resemble the central nervous system of the human body by lining it with various materials. They have local surface areas with the ability to perceive and actuate, as well as an interior communication array of wires. Additionally, it is feasible to print sensorics/actuating regions, micro-antennas, or communication cavities on the implant’s surface. A microcavity was made on a ZrO_2_ substrate, according to a novel study, and silver powder was then added and sintered into it. Silver baselines of high quality and low resistance were produced using 3D printing techniques [82].

### 3D printed scaffolds for bone regeneration

Patients experiencing bone fractures from various reasons, such as tumors and automobile accidents, always need to have their bones replaced. For instance, motor vehicle accident (MVA) causes craniofacial bone fractures, with the most common damaged sites being the zygomatic, nasal bone, and orbital floor [83]. These moderate sized fracture regions are good candidates for 3D printing. Because they offer a setting for cell adhesion, proliferation, and differentiation, scaffolds are crucial [84]. Connectivity between pores of appropriate size is critical for the transport of nutrients/metabolites and the growth of bone tissue. However, because high porosity usually reduces the mechanical stability of the scaffold, there are conflicts between biological and mechanical requirements. Therefore, the proper balance between these two standards must be considered when designing maxillofacial surgery scaffolds [62, 85–87]. What’s more, optimized porous structure plays an important role in 3D printed porous bone scaffolds suitable for the human body. There have been myriad attempts to manufacture scaffolds for osseous surgery with the purpose of promoting alveolar bone regeneration [88]. A detailed review on biocompatibility has been provided in above. Here we put emphasis on the mechanical properties of scaffolds.

A large number of zirconia composites and new types of surface treatment methods have been proposed in order to improve their biocompatibility. Studies demonstrating the preparation of the porous ZrO_2_/CS composite scaffold using DLP technology revealed that as the CS content increased, the aggregated CS connected with one another and gradually formed a stable support structure, increasing the compressive strength of the composite scaffold [66]. The production of this material by hydrothermal method has been also studied [89]. The 3D printed support is coated with Zn-HA/glass composite coating on glass impregnated zirconia (ZC). The compressive strength of ZC supports is 20% higher than that of zirconia (Z) supports [68]. 10CeTZP-Al_2_O_3_ porous mechanical casting structure shows good mechanical properties and aesthetic properties, which can be employed for the fabrication of bone scaffolds [90]. Mechanical properties have been conducted on zirconia and β -TCP compounded with PA 12 composite. Due to the addition of PA 12, the hybrid ceramics’ tensile modulus and impact strength are enhanced. Additionally, the new composite materials’ cell viability has been dramatically improved [91]. As for the structure of the scaffold, research has shown that diamond lattice unit (DIA) is a good choice for vascular growth, nutrient transport, and bone formation [92].

However, the printing method without scaffolds has been proposed called Kenzan method. This biological printer can effectively pick up the sphere and transfer it to the printing stage of a single complete layer, reducing the printing time of large organizational structures. By accurately transferring the ball bead to the printing surface, it is easy to build customized tissue on the needle [93]. This method is likely to become the future development direction.

Maxillary segments have been stabilized using 3D printed zirconia ceramic miniplates and screws after a Le-Fort I advancement surgery [94]. Compared with titanium implants, it has superiorities such as excellent biocompatibility and tissue integration without producing artifacts in CT or MRI scans, but also has disadvantages of high fracture risk. In addition, the three-dome space-maintaining barrier can be one of the directions for future experiments. It is suitable as an experimental tool to evaluate the possibility of using the designed barriers in dentistry and orthopedics to promote the formation of new bone and determine their space and time dependent limitations [71].

## Challenges of 3D printing in dental materials

The technologies of 3D printing zirconia have brought about progressive performance for complex and precise dental materials and structural design, which is unmatched by traditional subtractive methods. They have been explored in numerous territories of dentistry. Nevertheless, the slurry ratio of raw materials, porosity and agglomerates in the finished products, the connection between layers, as well as the unpredictable shrinkage rate, stand out as issues in the zirconia ceramic AM fabricating processes that still need to be resolved. Large-scale research efforts should be put on zirconia’s 3D printing technology.

### Raw materials and slurry preparation

At present, the application technologies of 3D printing in dentistry are mainly based on slurry. Seeking for an appropriate ratio of powder and liquid is extremely critical for 3D printing. The high ratio of powder to liquid contributes to lower pores and agglomerates on the product. Nevertheless, if the ratio is too high, it will count against the flowability and rheology of slurry and make it easier to settle. Typically, solid loads below 50% result in ZrO_2_ parts with low density, significant shrinkage, and defective pieces [95–97]. On the other hand, the slurry ratio determines the curing qualities. The critical exposure energy, penetration depth, and dispersion coefficient are the key determinants of the slurry’s curing properties. The quantity of photo initiator allows for adjustment of the critical exposure energy. The physical characteristics of the ceramic powder and the pre-mixed resin itself are related to the penetration depth and scattering coefficient.

### Porosity and agglomerates

The zirconia slurry used for printing dental products has a high viscosity. Air bubbles may consequently get entrapped during the printing process. If these air inclusions are located in the highly tensile stressed region of the specimens, they can spread across multiple layers and create worm-like pores, which eventually become fracture origins [34]. They can be distributed throughout various parts of the product, especially on the surface [8]. According to research, the porosities are classified into cavities for shrinkage and gas holes. Through experimental verification and theoretical analysis, it was shown that the air conveyed by powder spraying injection into the molten pool was the primary cause of the creation of pores [98]. Understanding the causes of pores is more helpful to reduce the generation of pores in the printing process. Porosity will also affect the accuracy of materials according to a recent study [59]. However, the porosity is still difficult to accurately be controlled during the fabrication of implants or scaffolds [43].

Agglomerates are remnants of the powder processing. These flaws can act as stress concentrators and lead to the failure of the specimens when loaded. However, differences were found in the fracture initiations of DLP and SM specimens, which were mainly reflected in the proportion, location, and size of fracture initiating pores [8]. More printing parameters and sintering programs related to porosity and agglomerates should become future research hotspots.

### Inter-layer defects

In the process of 3D printing fabricating, layer-by-layer printing is always performed and bonding between layers occurs. In general, inter-layer bonding in 3D printed materials is weaker than intra-layer bonding from a mechanical standpoint. Thus, the inter-layer bond strength is considered as one of the key parameters to ensure stability of the structure [99]. Pores and agglomerates are more likely to appear at the joint of layers, which cause spaces between two layers always becoming the weak areas of the final products [34]. Therefore, it is necessary to optimize the printing parameters so as to make the interlink areas more inseparable.

### Optimization of 3D printed zirconia materials

**Fig. 5:**
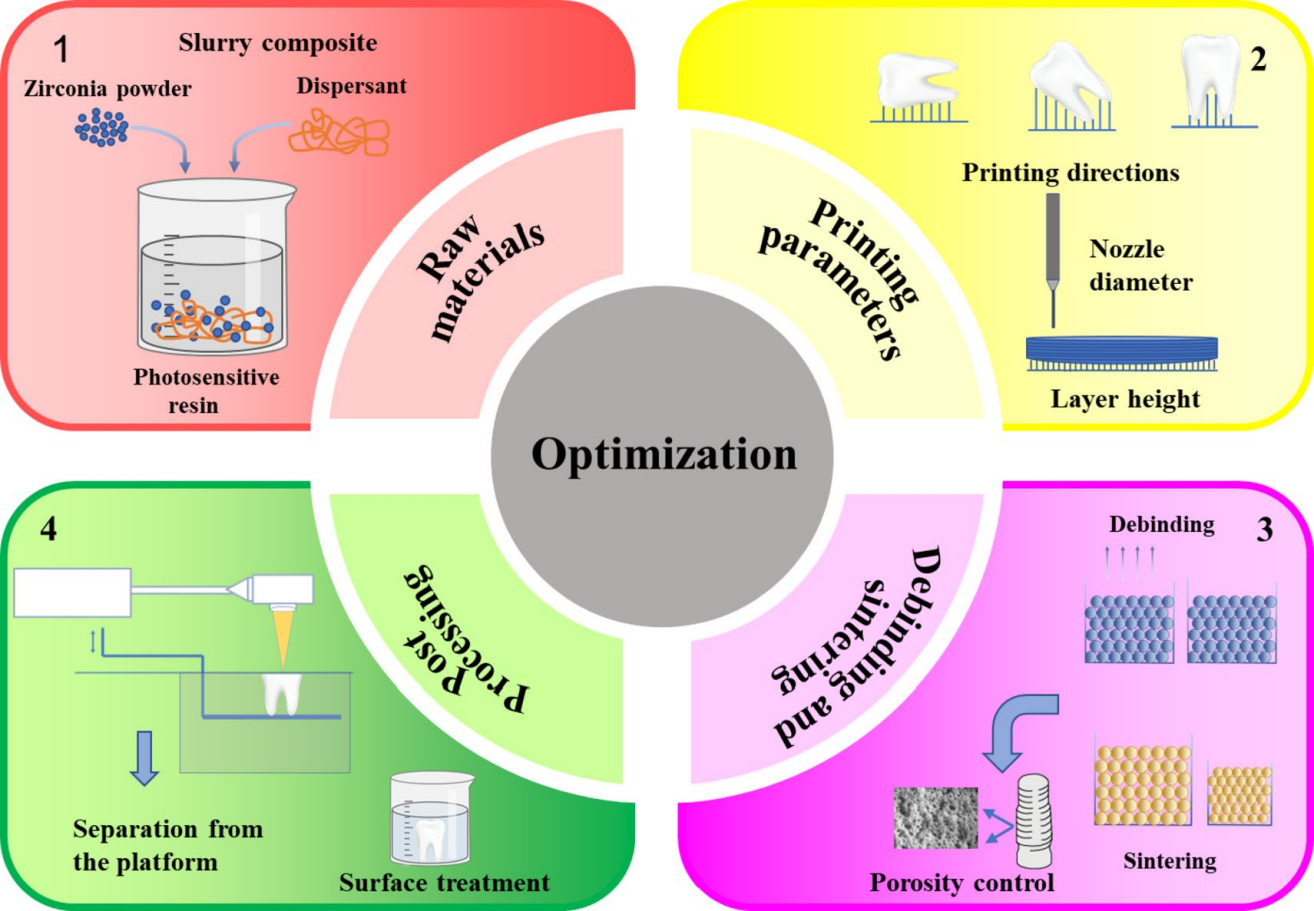
Optimization methods of 3D printed zirconia materials

The application of 3D printing zirconia ceramics in dentistry has enormous potentials. The issues mentioned previously have been the subject of extensive research efforts. 3D printing technology essentially involves the combination of the disciplines of engineering and materials science. There are various of factors affecting the quality of printed samples: raw materials, printing parameters, debinding and sintering as well as the separating procedures. Next, possible optimization methods around these four aspects will be elaborated (Fig. 5).

### Raw materials

The formulation of ceramic suspension is the first step in slurry-based 3D printing techniques, and the selection of raw materials is crucial as they determine the final performance of the material. Generally, it is determined by multitude aspects: the powder ratio, dispersant and slurry components. Numerous studies have been carried out to investigate the ideal slurry ratio, the characteristics of dispersing agents, and innovative forms of zirconia composites. Herein, the review focus on the recent progress of raw materials applied in dental 3D printing.

#### Balanced ratio of slurry

It is significant to find a balance between a large enough solid load and good flowability. The consistency of the slurry produced for printing determines the mechanical properties of the final products. Slurry using large particles and high dry matter content can ensure good and reliable density, bending strength and Weibull modulus. On top of that, as zirconia has a high refractive index and scatters light strongly, photopolymerization typically results in significant incident light scattering. As a result, the photopolymer’s cure depth is constrained, and anisotropic contraction takes place [100]. According to a study, the solid loading needs to be at least 40% in order to prevent flaws following debinding and sintering [96].The viscosity will significantly increase when the solid content exceeds 60% [101]. Researchers conducted experiments using a slurry containing 58% zirconia and obtained satisfactory mechanical properties [8, 35].

#### Dispersing agent in 3D printing zirconia

Dispersants play an important role in improving homogeneity of the slurry and reducing agglomerates on the final product. The solubility, potency and decomposition temperature of dispersant greatly affect the structure quality after washing and degreasing [102, 103]. Addition of Triton X-100 can reduce the surface tension of suspension [104]. 5 wt% DISPERBYK can effectively modify the zirconia powder for the purpose of preparing a homogeneous slurry [105]. Disperbyk-111 and PEGDA 250 have been proved to be smart decisions. Due to the spatial stability produced on the ceramic particles, 5 wt% of this dispersant (Disperbyk) produced low viscosity slurries with no discernible sedimentation [106]. More research should concentrate on the combination of different dispersants and adjust their concentration for better performance.

#### Alumina toughened zirconia

Researches on the mechanical properties of alumina toughened zirconia (ATZ) have been carried out [107]. The AM alumina toughened zirconia crowns show similar fracture resistance to traditional ones when fixed on the implant supported abutment [108]. In addition, the strength of prep increases after long-term aging [109, 110]. Some researchers provided possible explanations for this phenomenon. They discovered that the alumina distributed in the 3Y-TZP matrix could slow down the transformation by decreasing the TZP grains’ surface area in contact. Al_2_O_3_ has a larger elastic modulus than ZrO_2_, which reinforces the matrix’s pulling effect on the tetragonal phase. Furthermore, by inhibiting the interaction between the hydroxyl groups, the alumina segregation is a crucial element in obstructing the spread of transformation [28]. It can be assumed that ATZ is a promising implant material because it is less affected by phase transformation. Besides, it is noteworthy that a notable advantage of ATZ material is its light weight, so it shows great prospects in fabricating more comfortable oral materials.

#### 3D printed polymer-ceramic composites (PCCs)

Polymer infiltrated ceramics network (PICN) material is in progress for 3D printing to manufacture implants and scaffolds. By Combining polymer with zirconia, the mechanical strength of zirconia is closer to normal tooth tissue and bone tissue. Omitting the use of coating to achieve biocompatibility and better combining with bone tissue is the material goal. PICN samples have a larger capability to resist higher deformation than 50% infill ceramic scaffolds. Additionally, the presence of polymer helps to lessen the hardness for greater compatibility with natural teeth. The properties are desired for the use of zirconia material in the dentistry field. The polymer can be PA-12 and methacrylate [111, 112]. The mechanical qualities of the scaffold material are improved by the effective pentration of methacrylate copolymer. It has the strongest elongation resistance among similar materials. Moreover, it has the advantage of inhibiting bacterial reproduction [112]. Some additives are added like Si_3_N_4_ to avoid the problem of ultraviolet light scattering [113].

#### 3D printed zirconia composite containing silicon and carbon fiber

Zirconia has little biological activity and it is hard to form chemical or biological combination with bone. SiO_2_ interacts with calcium and phosphate ions in biological fluids to produce strong cell and tissue affinity. The study demonstrated that ZrO_2_ and SiO_2_ could combine in the liquid phase to generate the combination of ZrO_2_-SiO_2_. These ZrO_2_-SiO_2_ compounds exhibit good biological characteristics and are suitable for medical applications due to the fact that they can release silicate ions and encourage osteoblast development and differentiation. The strength and hardness of the experimental group with silane coupling agent added in the suspension are improved compared with those without silane coupling agent [62, 114]. Carbon black has been introduced into the raw materials, and an implant base was successfully printed, which acted as printing support for any overhangs [104]. In composites, the addition of carbon fiber can increase the toughness, which is essential for abutments and implants [115].

#### Other 3D printed zirconia materials

3D printed two-photon lithography zirconia materials have been studied by researchers. They emphasized that, in comparison to the acrylate system, shrinkage caused by the loss of organic matter was significantly decreased, and that zirconia seeds enabled the formation of micro ceramic in the pure and stable cubic phase. Additionally, even without any thermal treatment, the inclusion of nanoparticles affords a significant enhancement in the optical characteristics of the microfabricated structures [116]. As the concept of energy conservation gradually popularizes, myriad researchers focus on 3D printing with recycled zirconia powders. There are a great number of zirconia residues in materials applied in 3D printing. The purpose of saving raw materials can be achieved by recycling the remaining raw materials for reuse. However, the agglomerates in the recovered powders increase and the mechanical properties decrease when using the recycled powders. Researchers suggested that the original powder could be used for the restoration of posterior teeth, while the recycled powder could only be used for the anterior teeth [117].

### Printing parameters

#### Vat photopolymerization printers

Vat photopolymerization methods, such as SLA and DLP, are recommended for creating dense ceramic structures, as their applications have received the most attention [20]. For SLA and DLP, when the printing direction is horizontal, the dimensional accuracy and fracture toughness of the sample are obviously better than those of the vertical direction [38]. However, samples printed vertically have higher relative density, better semi-transparency, wettability and flexural strength. Besides, the printing direction influences the roughness of the surface of the final product at the same time [118]. To obtain the greatest mechanical properties, the best proportion of zirconia powder is different under different printing directions [118, 119].

Exposure energy and layer thickness also have a significant impact on product performance and final quality [120]. The quantity of photo initiators, particle size and shape, and exposure variables including wavelength, laser power, beam size and speed, exposure time and velocity are all factors that affect the resolution in the Z-direction, which is defined by the curing depth in each layer [121]. The Z-direction has the greatest variation [59]. Small layer heights make it easier to fully polymerize with each layer, which helps to avoid porosity and delamination problems at the layer borders. Defects in layer lines tend to lead to the DLP-printed zirconia’s failure. However, as the loading direction gets more favorable, the percentage of failures attributable to layer line faults diminishes [42]. It is necessary to further study the algorithm in which the ideal building angle is calculated before printing [57].

#### Material extrusion printers

The raw material used for material extrusion 3D printer needs sufficient liquidity. Moreover, printability enables sufficient strength to maintain stability of the printed structure immediately after deposition. Filament overlap ratio, printing speed and nozzle height affect the final performance of the material. Setting of appropriate overlap rate can effectively reduce printing defects, and thus improve the printing quality [122]. The layer height and printing speed have an impact on porosity. Low layer height and rapid printing yield the lowest surface roughness. The printing conditions affect the porosity and shrinkage percentage results. Low layer height and slow print speeds are correlated with the low porosity values [123]. The nozzle diameter affects the residual pore size on the surface of the material, thereby affecting the crack propagation resistance and the flexural strength of the material. The decrease in effective crack propagation resistance caused by the surface roughness of the curved surface and the large residual pore size on the fractured surface can be attributed to the excessive diameter of the extrusion nozzle, which leads to a loss of bending strength [124]. More studies should be conducted to find the standard nozzle diameter [124, 125].

#### Other printing techniques

Some novel printing methods have been developed to print zirconia dental materials. nanoparticle jetting (NPJ) is a unique technology that produces parts by spraying thousands of droplets. Products manufactured in this way have obtained satisfactory properties [126]. Wider application of 3D printing technology in clinical practice requires multidisciplinary cooperation. The optimal implant model with minimum fretting can be obtained by finite element simulation [127]. Combining two or more printing methods may become the future research directions [128]. The combination of two printing methods micro-stereolithography (PμSL) and direct ink write (DIW) has been used to print zirconia materials with specific porosity [129]. Ceramic injection molding (CIM) has low mechanical strength when used alone for manufacturing [130]. Free injection molding (FIM) is the product of combining CIM with 3D printing. The sample of this method has been studied, which showed that the problems of interlayer mixing defects and anisotropic shrinkage were avoided [131].

### Debinding and sintering parameters

Efforts have been put for improving the controllability of debinding and sintering process. The scheme is exactly essential and can greatly reduce the defects of the products. After curing, the dispersant, solvent, and polymer network in the green body are eliminated by the debinding procedure. The ceramic microparticles are then densified by the particle surface energy at a high temperature to create a dense ceramic component. Compared with conventional CAD/CAM manufactured products, the post-processing steps for 3D printed products are complicated. Sintering affects porosity and surface roughness. A great number of research were conducted to optimize the debinding and sintering procedures. Sintering actually affects the asynchronous densification phenomenon [132]. Researchers proposed two-stage sintering or slow temperature gradient sintering [32]. Additionally, the secondary shaping process can be improved with the use of high-temperature porous polymer molds to precisely regulate the structural geometry [60]. Using cold isostatic pressing (CIP) technology can reduce the defects between layers [99]. After 3D printing, annealing of the workpiece is helpful to improve the mechanical properties of the material. Compared with the unannealed composite, the thermal and mechanical properties of the annealed composite are improved in terms of the heat resistance and compressive strength [77]. Study showed that the grain size of the material rose with the increase of sintering temperature [133]. Besides, for zirconia with different transmittance, the optimum sintering temperature is different [133].

### Separation of sample from printing platform

The primary layer used in 3D printing, which must be removed once the body is constructed, fixes the green bodies to the building platform [134]. The green body deforms a tiny bit plastically upon removal. This results in the primary layer delamination at the margins closest to the construction platform, and this delamination persists even after sintering. But to a lesser level, delamination and edge damage also happen at the edges across from the building platform. One possibility of layering on this side is the formation of temperature gradients during sintering process [34]. In order to solve this problem, a novel method was proposed called Submersion-Light three-dimensional slurry printing method. The bottom-up methodology is the opposite of this one. The construction platform, which ordinarily goes up and down layer by layer, remains stabilized. In contrast, the light source, which ordinarily moves steadily up and down layer by layer is lowered into the vat rather than just relocated. What’ more, when printing products with complex shapes, the auxiliary supports may help regarding to this issue [31].

## Conclusions and prospective

Zirconia is a crucial ceramic material in dentistry, and in recent years, academics have been more and more interested in the creation of zirconia items using additive manufacturing. Digital processing in the whole process of prosthodontics will prove to be the development trend in the future [135]. This review recapitulates recent developments in additive manufacturing of zirconia ceramic materials and related applications in the field of dental materials. More significantly, it offers an analysis on the challenges and optimization methods of AM dental materials.

A great effort has been devoted to evaluate the basic properties of AM zirconia materials. With regard to the microstructure, AM and SM products have almost the same phase composition. The mechanical properties of AM products are usually slightly inferior to SM ones but there are usually no significant differences between them. It is noteworthy that the accuracy of AM zirconia materials can reach the clinical standard. They have extraordinary biocompatibility, which make them promising in oral implant and scaffold fields. Dental prostheses, implants, and maxillofacial surgical components are the typical examples of AM-produced zirconia materials that have been demonstrated in this review.

Though tremendous progress has been made regarding AM zirconia materials, it is not as developed compared to the AM of metal and polymer materials. Raw materials, printing parameters, and improving the mechanical and accuracy qualities of AM-processed zirconia products are still major issues. Each AM technique offers benefits and drawbacks specific to the production of dental zirconia materials. Seeking out a practical and controllable AM process to create dental zirconia parts with outstanding characteristics remains a significant problem. Hence, future research should pay more attention to these challenges AM zirconia faced.

The content above also emphasizes a series of methods for improving 3D printing zirconia technology. The AM techniques used to fabricate zirconia dental materials include SLA, DIP, MEX, and ink-jet printing, for which SLA and DLP have been the mainstream. Additionally, attempts have been made to use binder jetting and 3D gel printing as AM processes. For the slurry, a balance of powder and liquid has been explored. Several new methods were brought about to improve the debinding, sintering and other post processing procedures. Combination of different AM methods or combination of AM and SM methods should be further searched. 3D printing function graded materials might be new research hotspot [136, 137]. As for implantology, the structure can exhibit transition from a dense inner zirconia to a bioactive and soft material in the outer region in order to improve the stress distribution of cortical bone, bone trabecula and soft tissue [138]. To make items with different colors conceivable, novel ceramic printing techniques and equipment need also be developed for better aesthetics and color matching [139–142]. By applying additional modeling and simulation techniques into the investigation of AM of zirconia ceramics, the best strategy to fabricate zirconia components with superior performance is anticipated to be derived.

On account of the review, it is noteworthy that significant progress has been made in terms of biocompatibility of AM zirconia dental materials. However, porosity and aggregates occur because there is no consensus over the ideal ratio of powder to liquid for raw materials. Furthermore, AM technology and manufacturing parameters are the source of interlayer faults. As a result, it seems hard for AM to create dental zirconia materials with superior mechanical qualities and accuracy better than SM ones. Wherein accuracy is the most imperative issue. The clinical application of AM zirconia will become feasible upon the resolution of the aforementioned difficulties. To accomplish the perfect target in dental materials, an efficient and manageable AM technique workflow should be further investigated. Additionally, more clinical researches on the accuracy and mechanical characteristics of AM zirconia materials should be conducted.

## Data Availability

All the data and materials supporting the conclusions were included in the main paper.

## Abbreviations

AM: Additive manufacturing
ATZ: Alumina toughened zirconia
BMP-2: Bone morphogenic protein-2
CAD/CAM: Computer aided design/computer aided manufacturing
CIM: Ceramic injection molding
CIP: Cold isostatic pressing
CS: Calcium silicate
DIW: Direct ink write
DLP: Digital light processing
DPCs: Dental pulp cells
ECM: Extracellular matrix
FIM: Free injection molding
HAP: Hydroxyapatite
HBD3: Human beta defensin-3
hMSCs: Human mesenchymal stem cells
ISO: Organization for Standardization
LCM: Lithography-based ceramic manufacturing
LTD: Low temperature aging
MPSR: Modified proportional specimen resistance
NPJ: Nanoparticle jetting
PCC: Polymer-ceramic composites
PCL: Polycaprolactone
PFZ: Porcelain fused zirconia
PICN: Polymer infiltrated ceramics network
PLA: Polylactic acid
PSR: Proportional specimen resistance
SLA: Stereolithography
SM: Subtractive manufacturing
Y-TZP: Yttria tetragonal zirconia polycrystal
3D: Three-dimensional
